# Voltamperometric Discrimination of Urea and Melamine Adulterated Skimmed Milk Powder

**DOI:** 10.3390/s120912220

**Published:** 2012-09-05

**Authors:** Astrid Hilding-Ohlsson, Jonathan A. Fauerbach, Natalia J. Sacco, M. Celina Bonetto, Eduardo Cortón

**Affiliations:** 1 Biosensors and Bioanalysis Lab, Facultad de Ciencias Exactas y Naturales, Universidad de Buenos Aires (IQUIBICEN, CONICET), CABA 1428, Argentina; E-Mails: astrid@qb.fcen.uba.ar (A.H.-O.); natysacco@yahoo.com.ar (N.J.S.); celinatt@yahoo.com.ar (M.C.B.); 2 Departamento de Química Orgánica, Facultad de Ciencias Exactas y Naturales, Universidad de Buenos Aires (CIHIDECAR, CONICET), CABA 1428, Argentina; E-Mail: kempetai@gmail.com

**Keywords:** f-PCA, KNN, milk adulteration, voltammetry, rapid screening methods

## Abstract

Nitrogen compounds like urea and melamine are known to be commonly used for milk adulteration resulting in undesired intoxication; a well-known example is the Chinese episode occurred in 2008. The development of a rapid, reliable and economic test is of relevance in order to improve adulterated milk identification. Cyclic voltammetry studies using an Au working electrode were performed on adulterated and non-adulterated milk samples from different independent manufacturers. Voltammetric data and their first derivative were subjected to functional principal component analysis (f-PCA) and correctly classified by the KNN classifier. The adulterated and non-adulterated milk samples showed significant differences. Best results of prediction were obtained with first derivative data. Detection limits in milk samples adulterated with 1% of its total nitrogen derived from melamine or urea were as low as 85.0 mg·L^−1^ and 121.4 mg·L^−1^, respectively. We present this method as a fast and robust screening method for milk adulteration analysis and prevention of food intoxication.

## 1. Introduction

Dairy products have high nutritional value and are consumed all around the World, playing a fundamental role in international commerce and giving milk a great economic importance. For this reason they are vulnerable to several types of adulteration aiming at maximizing unscrupulous companies' profits [1]. Therefore, it was necessary to stipulate regulatory standards against food frauds and develop methods or tests to detect alterations and adulterations. Particularly, adulteration of milk with cheaper and sometimes toxic chemicals is a matter of serious concern. “Synthetic milk” is used to hide milk adulterations [2,3]; it is a mixture of components that imitates the composition and chemical characteristics of natural milk. Compounds like urea or melamine are usually added to “synthetic milk” to elevate its nitrogen content and mimic a high protein concentration. Both compounds have been found in dairy products and animal food as adulterants [4]. Food protein content is normally estimated by total nitrogen quantification using the Kjeldahl method [5]. This method cannot distinguish between nitrogen from protein and non-protein sources. Because of that, compounds as urea or melamine cannot be detected and are used as adulterants. Urea is an end product of nitrogen metabolism and a normal constituent of milk. It is usually found between 180 and 400 ppm and constitutes about 55% of the non-protein nitrogen compounds [2,6,7]. The cut-off concentration for urea in milk is normally set at 700 ppm [8]. The presence of urea in milk above this limit could cause severe health problems for humans such as indigestion, acidity, ulcers, *etc.* [3]. On the other hand, melamine (C_3_H_6_N_6_; 1,3,5-triazine-2,4,6-triamine) is an organic compound, slightly soluble in water, often used to produce synthetic polymers [9]. It has high nitrogen content (67% by mass).

In 2008, high levels of melamine were detected in some infant formulas and other liquid or powdered milk products originated in China. Melamine levels in these products were as high as 2,500 ppm [10–12]. As reported by the Chinese Ministry of Health, over 290,000 infants had been affected by melamine-contaminated infant formula by the end of November 2008. More than 50,000 infants were hospitalized, and six deaths were confirmed. Ingestion of high melamine concentrations produces adverse health effects such as the formation of crystals in the urinary system. Many affected infants in the Chinese incident had stones, or calculi, in the kidney, urether or bladder. Because of the large potential health impact, the World Health Organization (WHO) and the Food and Agriculture Organization of the United Nations (FAO) convened an Expert Meeting. Many countries have now introduced limits for melamine in infant formula and other foods. A tolerable daily intake (TDI) in powder infant formula of 1 mg/kg of body weight and in other foods of 2.5 mg/kg of body weight would provide a sufficient margin of safety for dietary exposure to melamine (WHO). Consequently, several methods have been developed for melamine and urea sample pretreatment and detection in food based on ELISA, HPLC-MS, solid phase extraction, ultraviolet spectrum, Raman spectroscopy and ion chromatography [9,13,14].

Electrochemical techniques have various applications and have been widely used in food analysis [15–19] of products such as coffee, fruit juices and milk [20,21]. Voltammetry seems to have several advantages; the technique has been used extensively in analytical chemistry due to features such as its very high sensitivity, versatility, simplicity and robustness [22,23]. Moreover, voltammetry offers a wide range of different analytical possibilities, including cycling, stripping, and pulse voltammetry. In a voltamperometric determination, the current is registered as a result of the applied potential. The registered signal depends on the composition of the sample and both the presence and concentration of electro-active molecules. When using voltammetry in complex media, data interpretation is very cumbersome because the responses obtained are often complex and nonlinear due to the many different processes that may occur on the surface of the electrode [24]. Multivariate calibration methods have shown to be useful to extract this information [25]. Functional data analysis (fda) is a collection of statistical methods for numerical data varying over a continuum [26,27]. These techniques were originally designed for time-varying data, but were later extended and applied to different kinds of data. The analytical process generally starts by fitting, with or without smoothing, curves of some selected families to the data. In this work each voltammogram data were fitted to a set of 50 B-splines polynomials of degree five, which provided a smooth fit that maintained the main features of the curves and reduced noise. One of the advantages of applying fda methods is that it is possible to work with original data or its derivatives. Furthermore, there are versions of multivariate statistical methods for functional data, such as principal component analysis (PCA) to explore the variation between curves. In regular PCA a rotation of the original data is obtained from the eigenvectors (principal component) of the variance-covariance matrix and the ordered eigenvalues indicate the importance of each successive principal component in terms of explained variance. In functional PCA (f-PCA) the eigenvectors are replaced by eigenfunctions. The visualization of functional data as a rotated set of principal components often results in a clearer display of the main patterns of functional data variation. Principal components analysis (PCA) is one of the most common and versatile statistical method for data handling and projection, being widely use in food analysis [21,23,25,28–31] and more specifically in milk analysis [1,32,33]. PCA data matrix consists of the experiment results (potential *versus* current in our data-sets) interpreted as variables. A corresponding score plot shows the relation between the observations or experiments and they could be grouped for classification by a supervised method as K nearest neighbors (KNN).

The primary objective of our investigation is to develop a new and easy method for detecting melamine and urea adulterations in milk and thus contribute to food analysis and security. We present here cyclic voltammetry experiments performed with a Au working electrode followed by f-PCA data analysis and KNN classification. This paper reports a novel screening method to detect milk adulterations with urea and melamine looking forward to develop a rapid and economical detection method.

## 2. Experimental Section

### 2.1. Electrodes and Instrumentation

A working electrode (WE) was home built with an Au wire of electrochemical grade (99.999% purity). A conductive silver-epoxy resin was used to connect the wire to a brass piece, all included on an epoxy resin body (Dystaltec EP Systems, Buenos Aires, Argentina). The electrode surface was observed with a stereomicroscope (Nikon SMZ645) in order to check the absence of air bubbles around the metal wire. The metal wire exposed as WE had a diameter of 2 mm (Au). Three other WE were also built, by a similar procedure (dental amalgam, Pt, Ag). Au WE gave the best results when used to discriminate adulterated from non-adulterated milk samples in comparison to the other materials (data not shown). Therefore, this work is focused in Au WE experiments.

A standard three electrode system was employed in the cyclic voltammetry assays using commercial KCl saturated Ag/AgCl electrode (OAKTON^®^, Vernon Hills, IL, USA) as a reference electrode, a stainless steel helicoidally electrode (CrNi; DIN: 1.4310; resistivity 0.43 mΩ·cm^−1^, 1 mm diameter) as a counter electrode (CE) and the home built WE. Cyclic voltammetries were performed with a commercial potentiostat (Gamry 300, Gamry Instruments Inc., Warminster, PA, USA).

### 2.2. Measurement Protocol

All measurements were performed after incubating 1 hour at 4 °C and subsequently incubating for 10 minutes at 40 °C to standardize experimental conditions and to assure melamine dissolution. Thereafter, the samples were gently homogenized and data acquisition was performed after 1 minute holding time in a thermostatic bath at 40 °C. Electrochemical tests were carried out in a single compartment three-electrode cell. After each measurement, electrodes were rinsed with distilled water (milliQ, 18.2 MΩ), and the planar WE was mechanically mirror-like polished for 10 seconds with alumina (aluminum oxide) of 1 and 0.3 μm granulometry consecutively. Thereafter, WE and CE were sonicated (Ultrasonic cleaner 8848, Cole-Parmer, Vernon Hills, IL, USA) in ethanol:water (50:50) for 2 minutes. RE was stabilized in a saturated KCl aqueous solution. Finally, all electrodes were thoroughly rinsed with distilled water and used in the next measurement.

### 2.3. WE Characterization

The WE characterization was performed with a phosphate buffer solution between −800 and +1,300 mV and with a potassium ferricyanide solution between −800 and +1,500 mV. These experiments were done at room temperature following the general procedure described before (Section 2.2). A phosphate buffer solution (200 mM, pH 7) was prepared with 18.8 g·L^−1^ KH_2_PO_4_ (Mallinckrodt, Hobart, NY, USA) and 11 g·L^−1^ of Na_2_HPO_4_ anhydrous (Carlo Erba, Milan, Italy) in distilled water (Double Reverse Osmosis Water System, NSF/ANSI 61, CCK^®^, Taipei, Neihu, Taiwan). If necessary, NaOH or HCl was added to keep neutral pH. A potassium ferricyanide (K_3_[Fe(CN)_6_], Sigma-Aldrich, St. Louis, MO, USA) stock solution (100 mM) was prepared in buffer phosphate (100 mM, pH 7). For further experiments, the optimal conditions were set to a scan rate of 25 mV·s^−1^ and a potential window between −800 and +1,000 mV to linearly scan the Au WE *versus* the reference electrode. Cyclic voltammetries started and ended at −800 mV.

### 2.4. Composition of Adulterated and Non-Adulterated Milk Samples

Milk samples (*M*) were made with rehydrated powdered skimmed milk of two different brands: *La Serenisima*^®^ (Buenos Aires, Argentina) and *Sancor*^®^ (Cordoba, Argentina) with distilled water (milliQ, 18.2 MΩ), as indicated by manufacturer's instructions (100 g·L^−1^). Replicated samples were prepared independently for quintuplicate (*La Serenisima*) or quadruplicate (*Sancor*).

“Synthetic milk” samples were based on an aqueous solution of distilled water (milliQ, 18.2 MΩ) containing 49 g·L^−1^ of sucrose (Mallinckrodt, Buenos Aires, Argentina), 3 g·L^−1^ of NaCl (Biopack, Buenos Aires, Argentina), and a nitrogen compound either urea (12.14 g·L^−1^, Ciccarelli, Santa Fe, Argentina) or melamine (8.5 g·L^−1^, Sigma-Aldrich). The components of the “synthetic milk” listed above mimics some native components found in bovine milk, so their concentration resembles the concentration of their respective components. These samples have the same nitrogen molar concentration than bovine milk. “Synthetic milk” samples were named as urea “synthetic milk” (*USM_0_*) or melamine “synthetic milk” (*MSM_0_*) depending on which nitrogen compound was used to prepare them. All samples were prepared daily. Melamine is slightly soluble in water so *MSM_0_* was prepared with hot distilled (milliQ) water (80 °C) and maintained at 40 °C to increase melamine solubility.

Adulterated milk samples were prepared by adding different proportions (20, 10, 5, 2 or 1%) of *USM_0_* or *MSM*_0_ to *M* to simulate series of gradual adulterated samples (1 to 5, see Table 1). Urea concentration ranged between 2.43 and 0.12 g·L^−1^ and melamine concentration ranged between 1.7 and 0.085 g·L^−1^ (Table 1). Conductivity (Digital Conductivity Meter, Curtin Matheson Scientific Inc., Houston, TX, USA) and pH (pHmeter, Hanna Instruments, Woonsocket, RI, USA) were kept at 4.7 mS·cm^−1^ and 6.80, respectively, to avoid electrochemical effects due to deviations from milk standard values of conductivity and pH.

### 2.5. Multivariate Data Analysis

The sensor outputs (cyclic voltammograms) collected by the potentiostat were imported to a personal computer and the statistical analysis was performed with R software (R Development Core Team) [34]. Voltammograms were recorded between −800 and 1,000 mV and data less than 400 mV was not included for PCA. Each voltammogram was fitted to a set of 50 B-splines polynomials of degree five, which provided a smooth fit that maintained the main features of the curves and reduced noise. Functional data analysis (fda) methods have been applied to original data and to its first order derivatives. The variation between curves has been explored by functional principal component analysis (f-PCA). PCA has been complemented with K nearest neighbors (KNN) analysis to classify milk samples into different levels of adulteration (no adulteration, low and high adulteration). The no-adulteration level was confirmed by non-adulterated milk samples. The USM_low level contains adulterated milk with 1, 2 and 5% of *USM_0_* and MSM_low level contains adulterated milk with 1, 2 and 5% of *MSM_0_*. The USM_high level contains adulterated milk with 10 and 20% of *USM_0_* and MSM_high level contains adulterated milk with 10 and 20% of *MSM_0_*. A “leave-one-out” strategy was applied to validate the KNN classifier.

## 3. Results and Discussion

### 3.1. Au Cyclic Voltammetries Characterization with Phosphate Buffer and Ferricyanide

**Figure 1.**
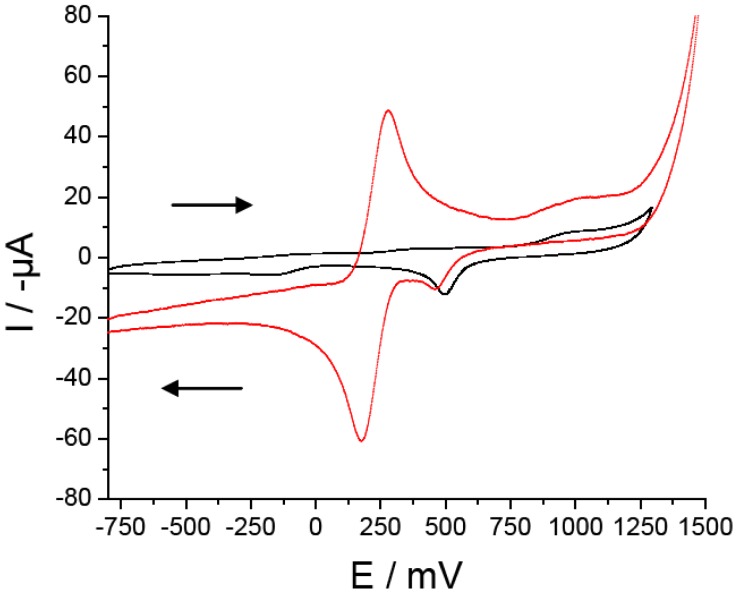
Au working electrode characterization by cyclic voltammetry in phosphate buffer with ferricyanide (red curve) and phosphate buffer (black curve). Scan rate: 25 mV·s^−1^. The scan direction is indicated by a black arrow. Ordinate shows negative current (I) values (−μA).

Au WE was characterized in phosphate buffer and in phosphate buffer with ferricyanide solutions (Figure 1). The scan direction is indicated by a black arrow. The upper scans indicate the oxidation of a substance on the surface of the WE resulting in a rapid rise of the anodic wave until the peak potential is reached.

When the scan direction was reversed towards negative potential the cathodic part of the wave (lower trace) was drown showing the reduction reactions. The phosphate buffer solution (black curve) showed no anodic peaks. The scan direction was reversed towards negative potential at +1,300 mV showing one small cathodic peak at Epc = +503 mV whose origin has not been determined, but is related to some compound/s present in the phosphate buffer. When the phosphate buffer with ferricyanide solution was scanned (red curve) one anodic peak appeared (Epa = +282 mV) corresponding to the oxidation of ferrocyanide to ferricyanide. The scan direction was reversed towards negative potential at +1,500 mV drawing the cathodic part of the wave and two cathodic peaks were seen. One peak around +176 mV corresponding to the reduction of ferricyanide to ferrocyanide and another at +468 mV with a smallest current which has a similar Epc than the phosphate buffer cathodic peak.

### 3.2. Au Cyclic Voltammetries in Adulterated and Non-Adulterated Milk Samples

**Figure 2.**
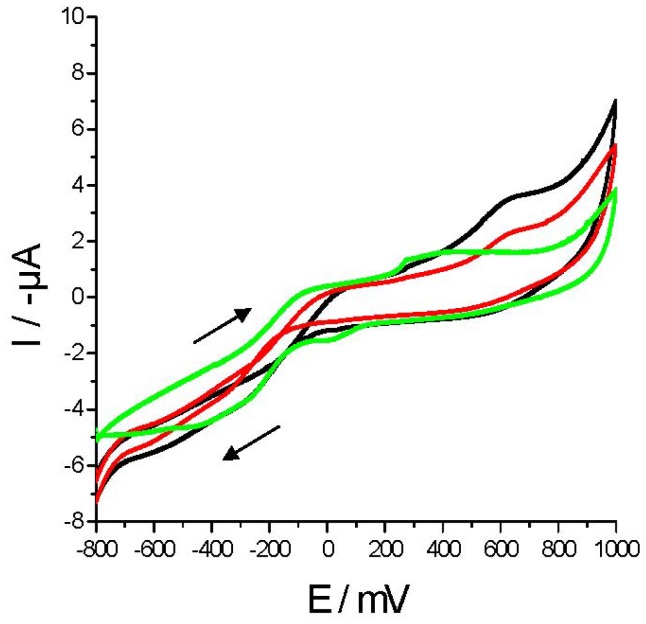
Cyclic voltammetries performed with Au WE over urea adulterated and non-adulterated milk samples: *M* (black curve); *USM_0_* (green curve) and *USM_1_* (red curve). Adulterant concentrations were 12.14 g·L^−1^ (*USM_0_*) and 2.43 g·L^−1^ (*USM_1_*) of urea. The scan direction is indicated by a black arrow.

**Figure 3.**
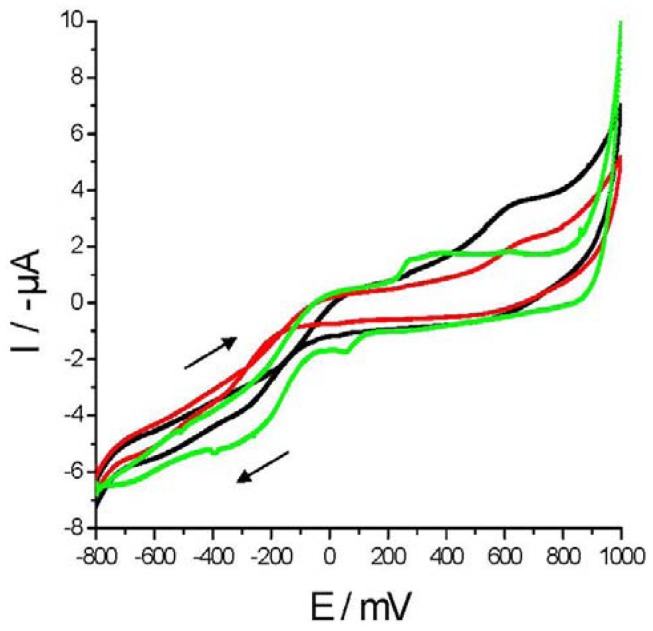
Cyclic voltammetries performed with Au WE over melamine adulterated and non-adulterated milk samples. *M* (black curve); *MSM_0_* (green curve) and *MSM_1_* (red curve). Melamine concentrations were 8.5 g·L^−1^ (*MSM_0_*) and 1.7 g·L^−1^ (*MSM_1_*) of melamine. The scan direction is indicated by a black arrow.

Cyclic voltammetries in milk and adulterated milk samples (with urea or melamine) from *La Serenisima* brand were performed (Figures 2 and 3) with Au WE. When *M* samples (black curve) were scanned only one anodic peak (Epa) appeared around +648 mV (Ipa = 1.65 μA). In Figure 2 control milk (*M*), melamine synthetic milk (*MSM_0_*) and one adulterated milk sample (*MSM_1_*) voltammograms are plotted. *MSM_0_* (green curve) showed one anodic peak at around Epa of +292 mV (Ipa = 0.51 μA) and one cathodic peak at around Epc = +5.6 mV with a similar current value (Ipc = 0.49 μA) which is not present in *M* neither in *MSM_1_*.

On the other hand, the peak that appeared in *M* at Epa = +648 mV is present in *MSM_1_* showing a current reduction (Ipa = 1.06 μA) and it is not present in *MSM_0_*. In Figure 3 *M*, urea synthetic milk (*USM_0_*) and one adulterated milk sample (*USM_1_*) voltammograms are plotted. The *USM_0_* voltammogram (green curve) showed one anodic peak at around Epa of +281 mV (Ipa = 0.68 μA) and one cathodic peak at around Epc = +40 mV (Ipc = 0.84 μA) which are not present in *M* nor in *USM_1_*. In contrast the anodic peak of *M* is also present in *MSM_1_* showing a current reduction (Ipa = 1.08 μA) but it is not present in *MSM_0_*. From these figures it is noticed that although adulterated milk samples voltammograms have the same shape than *M* voltammograms, the addition of “synthetic milk” results in a decrease of current values. As the concentration of “synthetic milk” decreased, the greater is the similarity between its cyclic voltammetries and the voltammetries of M.

Other authors [35] using a glassy carbon electrode *versus* Ag/AgCl_sat_ and performing cyclic voltammetries in milk obtained only one anodic peak located around +732 mV but no cathodic peaks at all. Even though the reacting species present in the samples are not known, in 1991, Jawad *et al.* [36] using a carbon paste electrode *versus* Ag/AgCl_sat_ and performing cyclic voltammetries in milk postulated that uric acid is the responsible for an anodic peak at +845 mV.

Cyclic voltammetries of *M* and all the adulterations with *USM_0_* and with *MSM_0_* (1 to 5, see Table 1) made with *La Serenisima* milk were analyzed by PCA. The analysis of the PCA-rotated data showed that the major amount of the variance of the data is represented in the section between +400 mV and +1,000 and between +1,000 mV and +400 mV which contains the anodic peak around +670 mV present in *M* and adulterated milk samples. The application of functional data analysis allowed us to analyze the whole curves instead of breaking them in individual sample points as in regular PCA. Therefore, these sections of the voltammograms were selected to performed f-PCA and the sections before +400 mV has not been included in the analysis.

**Figure 4.**
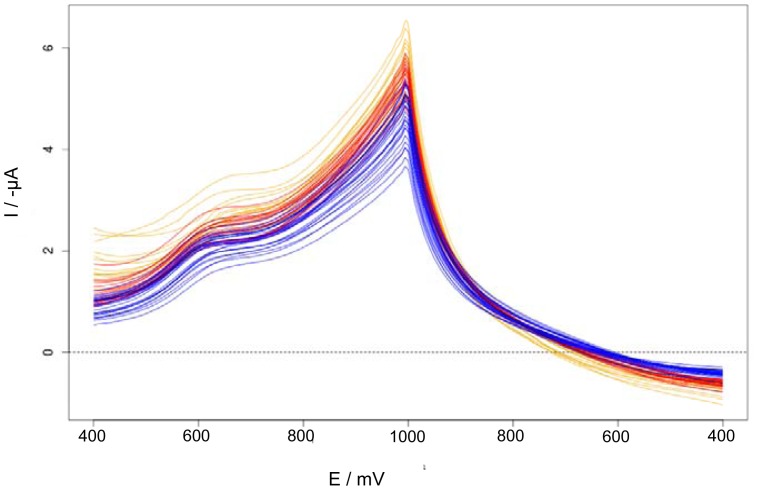
Cyclic voltammetries sections obtained with Au WE in different adulterated and non-adulterated milk samples. *La Serenisima* milk (*M*) samples (orange curves); *M* adulterated with melamine (*MSM*) (red curves); *M* adulterated with urea (*USM*) (blue curves). Adulterant concentrations ranged between 2.43 and 0.1214 g·L^−1^ for urea and between 1.7 and 0.085 g·L^−1^ for melamine (see Table 1).

**Figure 5.**
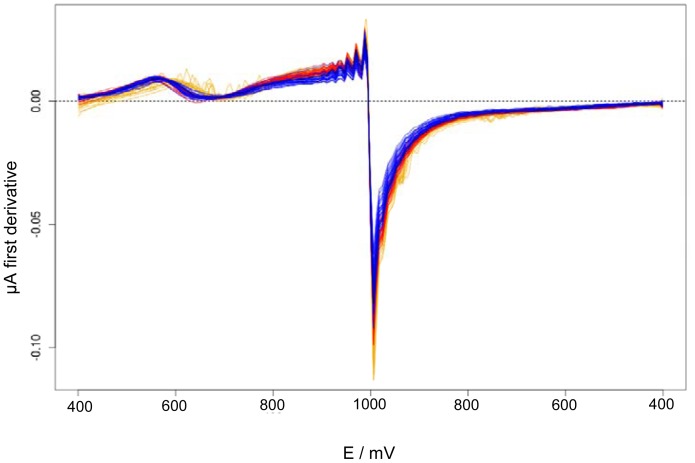
First derivative of the selected sections of cyclic voltammetries performed with Au WE over adulterated and non-adulterated *La Serenisima* milk samples. *M* (orange curves); *M* samples adulterated with melamine (*MSM*) (red curves); samples adulterated with urea (*USM*) (blue curves).

Figure 4 shows the selected cyclic voltammetries sections of all *La Serenisima* brand samples described in Table 1. Differences between the cyclic voltammetries of different samples (non-adulterated milk, urea adulterated milk and melamine adulterated milk) could be seen. Non-adulterated milk samples have bigger current values than the ones found for adulterated milk. The minor current values correspond to urea adulterated samples. Figure 5 shows the first derivative of the selected section of the cyclic voltammetries. In this figure the features are incremented and new peaks appeared.

Organic compounds like melamine or urea decreases current values in our cyclic voltammetries probably by “fouling” the electrode surface. There are interesting reports of the self-assembly of melamine structures on gold surfaces [37], also the adsorption of urea on gold surface has been suggested [38]. We consider that urea and melamine are responsible of most of the current decrement found in our voltammograms presented in Figures 2 and 3. Control experiments show that synthetic milk components (melamine, urea and sucrose) directly and independently added to milk samples decreases current values (data not shown). These results are in agreement with the results found in bibliography. Therefore, we conclude that each component present in “synthetic milk” contribute to milk adulteration changing its characteristics which are reflected in the decrement of current values.

### 3.3. F-PCA of Cyclic Voltammetries of Adulterated and Non-Adulterated Milk Samples

**Figure 6.**
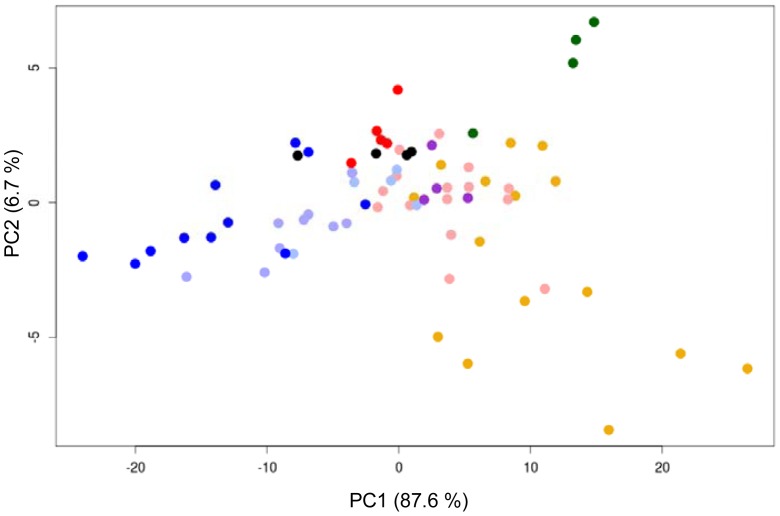
Functional PCA join results of original cyclic voltammetric data obtained with Au WE in different adulterated and non-adulterated milk samples. *La Serenisima M* ( 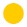); *MSM* ( 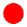); *USM* ( 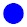); *Sancor M* ( 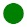); *MSM* (●); *USM* ( 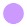). Adulterant concentrations ranged between 2.43 and 0.1214 g·L^−1^ for urea and between 1.7 and 0.085 g·L^−1^ for melamine. For *USM* and *MSM* a graduation in the color intensity represents the adulteration percentage from 1 to 20%, where increasing intensity corresponds to a higher adulteration.

**Figure 7.**
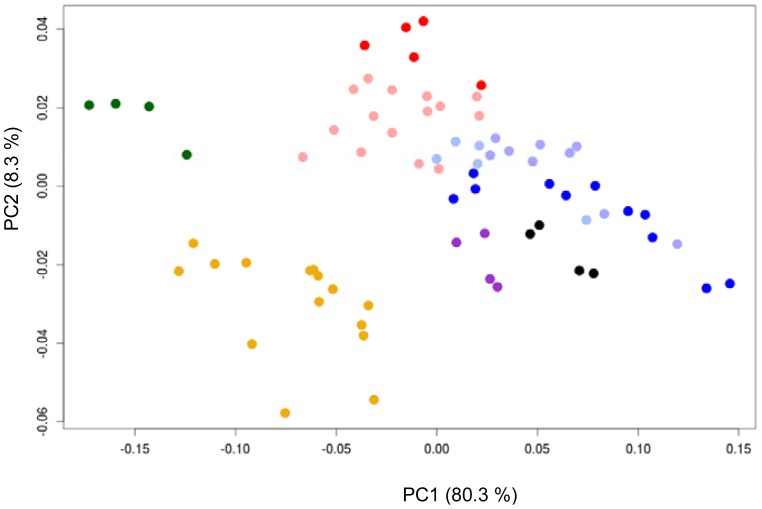
Functional PCA join results of first derivative of cyclic voltammetries obtained with Au WE in different adulterated and non-adulterated milk samples. *La Serenisima M* ( 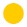); *MSM* ( 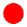); *USM* ( 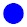); *Sancor M* ( 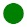); *MSM* (●);*USM* ( 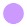). Adulterant concentrations ranged between 2.43 and 0.1214 g·L^−1^ for urea and between 1.7 and 0.085 g·L^−1^ for melamine. For *USM* and *MSM* a graduation in the color intensity represents the adulteration percentage from 1 to 20%, where increasing intensity corresponds to a higher adulteration.

Functional data analysis (fda) methods has been applied to original data (Figure 6) and to its derivatives (Figure 7) and the variation between curves has been explored by functional principal component analysis (f-PCA).

All adulterated samples could be separated correctly from control samples (*M*). Figure 6 shows the tendency of the original data to group in different places of the plot. These differences are enhanced with derivative data (Figure 7). A new milk brand (*Sancor*) was included in the analysis following the same procedure used for *La Serenisima* brand. For *Sancor* samples adulterated milk was represented by an in-between concentration of adulterant (*MSM_3_* and *USM_3_*). These adulterated samples are all plotted near *La Serenisima* adulterated samples and separate from all non-adulterated samples, both *La Serenisima* and *Sancor* (Figures 6 and 7). It is also possible to conclude that the percentage of adulteration has influence on the PCA response, confirming that the discrimination in the case of adulterated samples is caused by the ability of the sensor to detect the adulterants [16].

### 3.4. Classification of Adulterated and Non-Adulterated Milk Samples by KNN

f-PCA has been complemented with K nearest neighbors (KNN) analysis to classify milk samples into five different levels of adulteration (no-adulteration, low melamine or urea adulteration and high melamine or urea adulteration). A “leave-one-out” strategy was applied to validate the KNN classifier, for non-treated cyclic voltammetries (Table 2) and for first derivative of cyclic voltammetries (Table 3). Better results were found in the latter case (first derivative data), showing that we could discriminate non-adulterated milk samples from those adulterated with 20, 10, 5, 2 and 1% of *USM_0_* or *MSM_0_* as they were correctly classified into different groups. Each sample was correctly classified into its level (Table 3). The less adulterated samples that were able to discriminate from non-adulterated milk have a concentration of urea and melamine of 121.4 and 85 mg·L^−1^, respectively, which corresponds in both cases to adulterated milk with 1% of its total nitrogen from either adulterant.

The system was trained with *La Serenisima* milk and then a second batch of non-adulterated and adulterated samples from *Sancor* was used as a test set. When classification test were performed with first derivative data all the control samples from the test batch (*Sancor*) were correctly classified as non-adulterated, and none of the adulterated samples was assigned to the control class. All adulterated samples were classified as *USM* (high or low) irrespectively of the adulteration type.

The ability of the Au WE to discriminate the samples containing “synthetic milk” with urea or melamine is clearly noticed, as *M* samples are all correctly classified for both batches. These results suggest that this method is able to discriminate between milk and adulterated milk with “synthetic milk” containing different nitrogen compounds such as urea and melamine reaching a discrimination level of 1% of adulteration. Moreover, the fact that no sample pretreatment is needed, as the addition of extracting solvents, shaking, centrifugation, filtering, diluting, *etc.* [39–41] is an important advantage to be considered when a fast milk screening is needed and immediate decisions have to be taken by the quality controlling food authorities as occurred in China in 2008.

Since the sad Asian event, more analytical methods are available, as recently reviewed [41]; most of these sensitive and specific methods, based in HPLC-MS/MS can reach ppb melamine levels, but they are expensive and requires very competent technicians; both factors limits strongly their utility in developing countries, where our screening method could be very useful, in order to prevent intoxications.

## 4. Conclusions

Best results were obtained with the first derivative of a section of the cyclic voltammetries performed with Au WE in milk samples. f-PCA allowed us to differentiate simulated adulterated milk samples containing urea or melamine from non-adulterated milk samples. We could separate non-adulterated milk from adulterated milk samples with 20, 10, 5, 2 and even 1% of “synthetic milk” with urea or melamine by the correct classification of each adulteration in different groups (levels of adulteration). By this method, the less adulterated samples that were able to discriminated from non-adulterated milk corresponding to adulterated milk with 1% of its total nitrogen from either adulterant (urea or melamine). These samples have a concentration of urea and melamine of 121.4 and 85 ppm, respectively. Considering the maximum melamine concentration found in Chinese adulterated samples (2,500 ppm) the presented method proves to be useful to detect samples with a concentration 30 times lower that the aforementioned maximum. If we apply the TDI (2.5 mg·kg^−1^) established by WHO to a 75 kg adult it will allow a safe intake of 187.5 mg of melamine per day. Therefore our detection limit is enough to protect an adult whose daily ingest is up to 2.2 liters of milk. These results proves that the screening method presented in this work could provide a simple, rapid and inexpensive method able to detect real cases of milk adulterations and prevent food intoxication with melamine. In addition the urea concentration (down to 121.4 ppm) of milk samples containing “synthetic milk” with urea that were able to discriminate from non-adulterated milk, suggests that this method could be useful to detect urea adulterations given that the maximum concentration of urea allowed for milk is 700 ppm. The performance, low cost, reproducibility and simplicity of the fabricated working electrode and the electrochemical technique demonstrated the advantages of this method to detect adulterated milk samples in a rapid method that could be used to prevent acute intoxications.

## Figures and Tables

**Table 1.** Composition of adulterated and non-adulterated milk samples. *M*: Milk, *USM*: Urea synthetic milk, *MSM*: Melamine synthetic milk. Adulterated samples (1 to 5) were prepared by mixing a percentage of *M* and the correspondent “synthetic milk” (*USM_0_* and *MSM_0_*). The quantity of adulterant (urea or melamine according to each case) is indicated in g·L^−1^.

**Sample ID**		**% *M***	**% *USM***	**% *MSM***	**Adulterant (g·L^−1^)**

*M*	-	100	-	-	0
*USM*	0	-	100	-	12.14
	1	80	20	-	2.43
	2	90	10	-	1.25
	3	95	5	-	0.61
	4	98	2	-	0.24
	5	99	1	-	0.12

*MSM*	0	-	-	100	8.50
	1	80	-	20	1.7
	2	90	-	10	0.85
	3	95	-	5	0.43
	4	98	-	2	0.17
	5	99	-	1	0.085

**Table 2.** K nearest neighbor (KNN) confusion matrix constructed by “leave-one-out” procedure over f-PCA of non-treated original cyclic voltammetric data sections of *La Serenisima* adulterated and non-adulterated milk. Adulterated milk samples were grouped divided into high or low adulteration level. High level was composed by MSM1 and MSM2 or USM1 and USM2, respectively. Low adulteration group was composed by MSM3, MSM4, and MSM5 or USM3, USM4, and USM5, respectively. *M*: Milk.

**Class**	**M**	**MSM_High**	**MSM_Low**	**USM_High**	**USM_Low**
M	8	0	7	0	0
MSM_high	0	7	0	0	2
MSM_low	3	0	12	0	0
USM_high	0	0	1	9	1
USM_low	0	1	1	1	12

**Table 3.** K nearest neighbor (KNN) confusion matrix constructed by “leave-one-out” procedure over f-PCA of first derivative of cyclic voltammetries sections of *La Serenisima* adulterated and non-adulterated milk samples. Adulterated milk samples were grouped divided into high or low adulteration level. High level was composed by *MSM_1_* and *MSM_2_* or *USM_1_* and *USM_2_*, respectively. Low adulteration group was composed by *MSM_3_*, *MSM_4_*, and *MSM_5_* or *USM_3_*, *USM_4_*, and *USM_5_*, respectively. *M*: Milk.

**D Class**	**M**	**MSM_High**	**MSM_Low**	**USM_High**	**USM_Low**
M	15	0	0	0	0
MSM_high	0	7	2	0	2
MSM_low	0	2	13	0	0
USM_high	0	0	0	9	1
USM_low	0	0	2	1	12
